# An Inactivation Switch Enables Rhythms in a *Neurospora* Clock Model

**DOI:** 10.3390/ijms20122985

**Published:** 2019-06-19

**Authors:** Abhishek Upadhyay, Michael Brunner, Hanspeter Herzel

**Affiliations:** 1 Institute for Theoretical Biology, Charité—Universitätsmedizin Berlin and Humboldt University of Berlin, Philippstr. 13, 10115 Berlin, Germany; 2 Biochemistry Center, University of Heidelberg, Im Neuenheimer Feld 328, 69120 Heidelberg, Germany; michael.brunner@bzh.uni-heidelberg.de

**Keywords:** circadian clock, mathematical modeling, molecular switch, *Neurospora crassa*, glucose compensation

## Abstract

Autonomous endogenous time-keeping is ubiquitous across many living organisms, known as the circadian clock when it has a period of about 24 h. Interestingly, the fundamental design principle with a network of interconnected negative and positive feedback loops is conserved through evolution, although the molecular components differ. Filamentous fungus *Neurospora crassa* is a well-established chrono-genetics model organism to investigate the underlying mechanisms. The core negative feedback loop of the clock of *Neurospora* is composed of the transcription activator White Collar Complex (WCC) (heterodimer of WC1 and WC2) and the inhibitory element called FFC complex, which is made of FRQ (Frequency protein), FRH (Frequency interacting RNA Helicase) and CK1a (Casein kinase 1a). While exploring their temporal dynamics, we investigate how limit cycle oscillations arise and how molecular switches support self-sustained rhythms. We develop a mathematical model of 10 variables with 26 parameters to understand the interactions and feedback among WC1 and FFC elements in nuclear and cytoplasmic compartments. We performed control and bifurcation analysis to show that our novel model produces robust oscillations with a wild-type period of 22.5 h. Our model reveals a switch between WC1-induced transcription and FFC-assisted inactivation of WC1. Using the new model, we also study the possible mechanisms of glucose compensation. A fairly simple model with just three nonlinearities helps to elucidate clock dynamics, revealing a mechanism of rhythms’ production. The model can further be utilized to study entrainment and temperature compensation.

## 1. Introduction

The Earth’s rotation around its own axis gives rise to the 24-h day and night cycles. To anticipate these daily environmental changes in light and temperature, life in all its kingdoms has evolved a time-keeping molecular machinery [1,2,3]. In humans, disruption in the clock functioning due to jet lag, work shift, light at night, or other reasons increases the risk of diseases such as cancer, obesity, and sleep disorders [4,5,6].

In order to understand the basic clock mechanisms, we study a filamentous fungus, *Neurospora crassa* (*N. crassa*) [7]. Their natural habitats are soils, plants, trees, and food resources [8]. The ability of *N. crassa* to deconstruct and metabolize plant cell walls is crucial for environmental carbon and other nutrient cycling. There are also various potential biotechnological applications such as the production of biofuels. An example is the production of ethanol from xylose derived from plant dry matter biomass (lignocellulosic substrates). The xylan to ethanol pathway has been found to be clock regulated at each stage [9].

Mathematical modeling is useful to understand the *Neurospora* clock mechanism with multiple known feedback loops [10,11,12,13,14,15]. There are earlier models that considered only the core clock gene *Frequency* (FRQ) [10,11,12]. Recently, transcription factor White Collar-1 (WC1) has also been incorporated into the models [13,14,15]. In order to study glucose compensation, another feedback via Conidial Separation-1 (CSP1) [16] was included.

**Figure 1:**
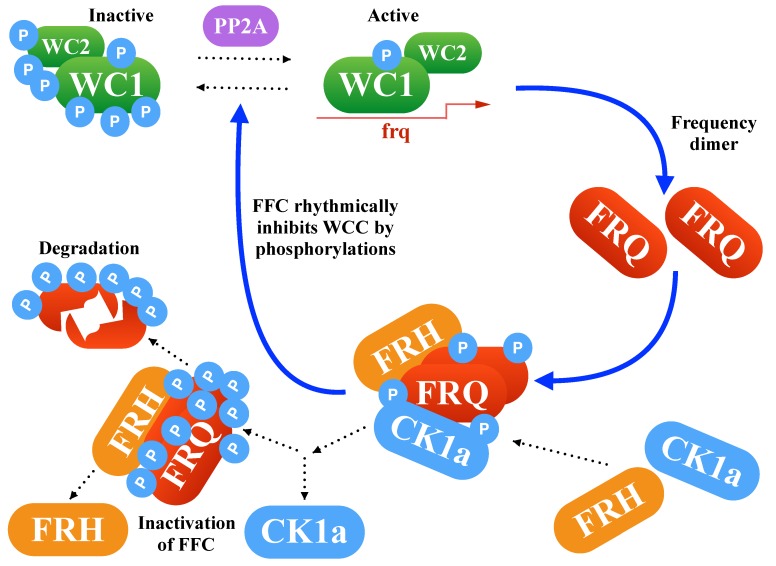
Core network of *Neurospora*’s clock: The delayed negative feedback via FRQ is controlled by complex formations and phosphorylations.

Figure 1 demonstrates the transcription-translation feedback loop, which consists of the inhibitory protein Frequency (FRQ), the activating transcription factor White Collar Complex (WCC), the FRQ stabilizer Frequency-interacting Helicase (FRH), and the Casein Kinase-1a (CK1a). Oscillations are based on delayed negative feedback. In *Neurospora*, clock gene *frequency* inhibits its own transcription after intermediate steps such as transcription, translation, dimerization, phosphorylations, and nuclear import. Moreover, stabilization of FRQ is governed by FRH and CK1a binding in forming the FFC complex. Later, FFC is involved in the inhibition of WCC-induced transcription [17,18,19].

The detailed mechanisms of the negative feedback are not well understood. For example, the inhibition of WCC via FFC might involve hyperphosphorylation, sequestration, and stoichiometric inhibition [20,21,22,23,24]. Moreover, it is not clear how the long delay is realized to obtain daily rhythms. In order to obtain self-sustained oscillations, nonlinearities are necessary.

The focus of our study is a deeper understanding of the underlying nonlinearities (“switches”) and an analysis of the possible intrinsic mechanisms of glucose compensation in the *Neurospora* clock. Thus, our model addresses two general questions: (1) What are the underlying switch mechanisms? (2) How can a steady period be maintained for varying environmental conditions such as glucose availability? A comprehensive analysis of our mathematical model reveals that an inactivation switch allows self-sustained rhythms in the *Neurospora* clock. Our model also suggests in-built glucose compensation mechanisms.

## 2. Results

### 2.1. Modeling the Core Clock Elements

The negative feedback loop involves an implicit delay consisting of frq transcription and translation, FRQ dimerization, CK1a binding, nuclear import, formation of the FFC complex, and FFCn interaction with WC1n. A positive feedback involves the nuclear export of FFC supporting WC1c translation [25,26]. It is well known that FRQ and WCC are phosphorylated at many sites. Rhythmic phosphorylation influences complex formation and stability [27,28]. In our current model, we took these phosphorylations implicitly into account via appropriate rate constants.

**Figure 2:**
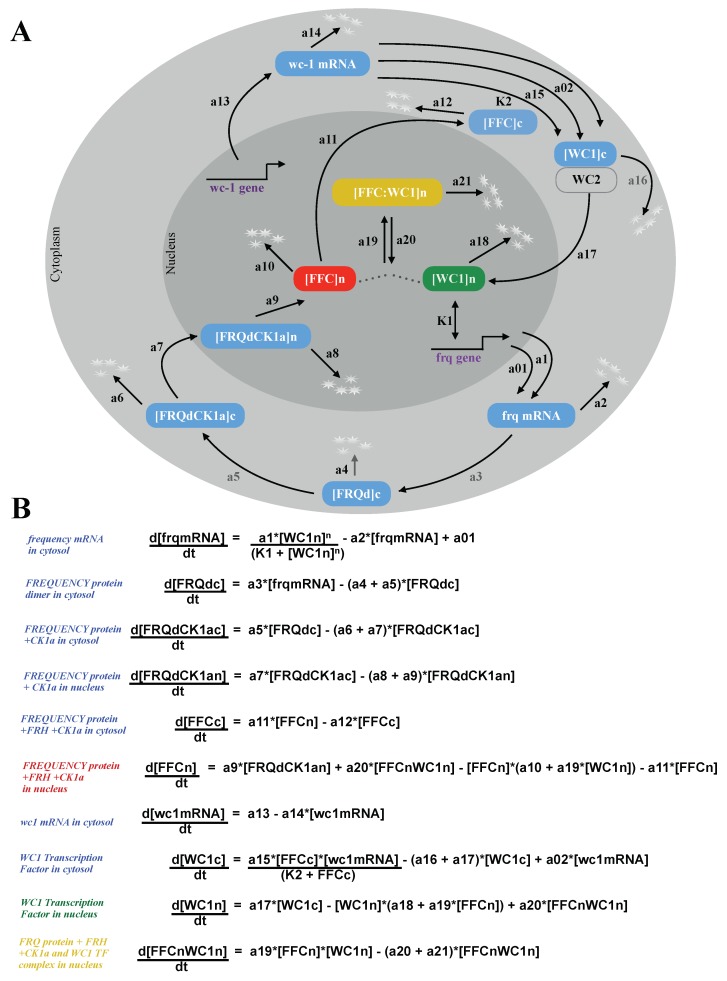
*Neurospora* circadian clock model: The wiring diagram of the model shows compartmentalization into nucleus and cytoplasm, turnover of frq and wc1 colored in blue, complex formations, and nuclear translocation. The core components of the inactivation switch are marked by green, red, and yellow throughout the paper (**A**). Ordinary differential equations with 10 variables and 26 parameters (**B**).

**Figure A1:**
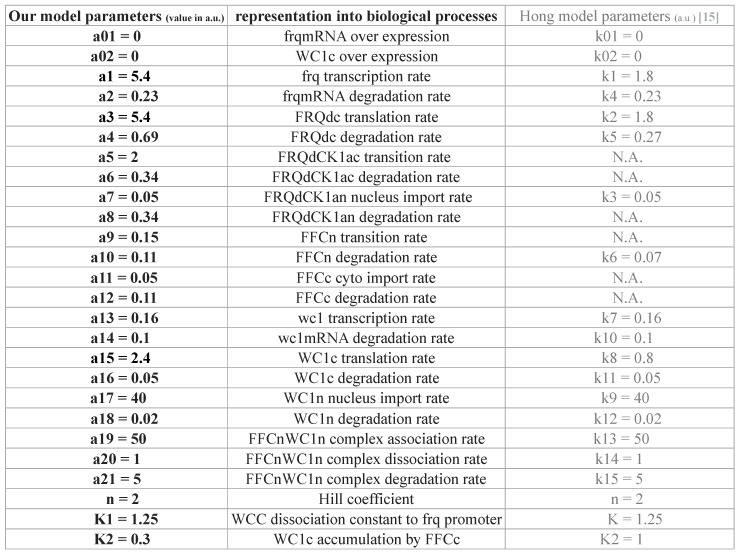
Kinetic parameters: A list of rate constants and their associated biological processes.

Figure 2A shows the turnover of frq, wcc, and associated negative and positive feedback loops. Using primarily linear kinetic terms, this scheme can be directly translated into a system of Ordinary Differential Equations (ODEs) [29,30,31,32]. These equations are presented in Figure 2B. The 10 time-varying concentrations are denoted with brackets, and the 26 kinetic parameters are enumerated as a01, a02, a1, a2, ..., a21, n, K1, K2 (Appendix Figure A1).

### 2.2. The Model Reproduces Self-Sustained Rhythms

The basic scheme of the model is adapted from a previous study [15]. In order to study the role of FRH and FFC, we introduced three additional variables [FRQdCK1a]c, [FRQdCK1a]n, and [FFC]c (see Figure 2). The corresponding five parameters (a4, a6, a8, a10, a12) were taken from the proteins’ half-lives in associated experiments [27,33]. In order to adapt expression levels, we increased the frq transcription rate (a1) and translation rate (a3, a15) (see Appendix Figure A1).

We kept most parameter values from the original model [15] (compare Appendix Figure A1). The remaining two parameters corresponding to the FRQdCK1a and FFC complex formation rates (a5 and a9) were adjusted to get a period of 22.5 h and robust limit cycle oscillations. Moreover, our model included a stronger positive feedback on cytoplasmic WC1 by FFC. We decreased the activation threshold from K2 = 1 in [15] to A2 = 0.3 in our model to strengthen the experimentally-verified positive feedback [25] (compare Appendix Figure A1).

Our resulting model consisted of 10 ODEs and 26 kinetic parameters. In order to get self-sustained rhythms, nonlinearities are necessary. Inspection of the equations in Figure 2B reveals that there were just three nonlinear terms: The transcription of the gene frq was modeled via a quadratic Hill function representing multiple WCC binding sites. The complex formation of FFC and WC1 was described by a bilinear term. The third nonlinearity modeled the positive feedback of the FFCc complex on WC1c translation as in the original model [15].

**Figure 3:**
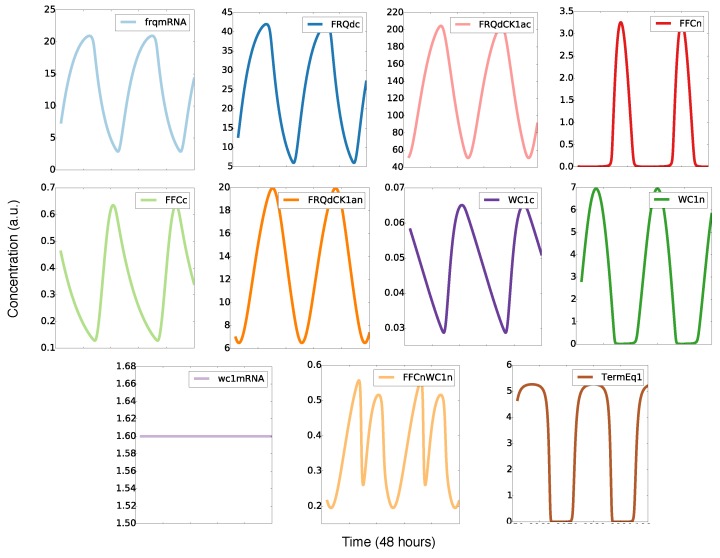
Simulated time series: There are sinusoidal and spike-like waveforms, harmonics, and a temporal switch (see the text).

Figure 3 shows simulations of our model for the default parameters listed in Appendix Figure A1. The oscillations of frequency mRNA and protein (blue) were almost sinusoidal, whereas the levels of the inhibitory complex FFCn (red) and the transcription factor WC1n (green) displayed spike-like behavior. The resulting FFCnWC1n complex (yellow) exhibited even two peaks during one circadian cycle. This observation illustrates that nonlinearities can generate pronounced “harmonics”, an example of ultradian rhythms. Harmonics have been described in large-scale transcription profiles in mouse [34,35] and recently also in *Neurospora* [36]. The final graph in Figure 3 represents the transcription of the frq gene. It is evident that there was a sharp ON/OFF switch of transcription. In a later section, we will relate this temporal switch to FFC-assisted inactivation of the transcription factor WC1.

### 2.3. Bifurcation Analysis of Our Model

The available kinetic data were not sufficient to determine all model parameters precisely. Moreover, external conditions can induce parameter variations. Consequently, we varied all 26 parameters in a comprehensive manner to explore the robustness of the model and to evaluate the role of the different kinetic terms along the lines of previous studies [37,38].

**Figure 4:**
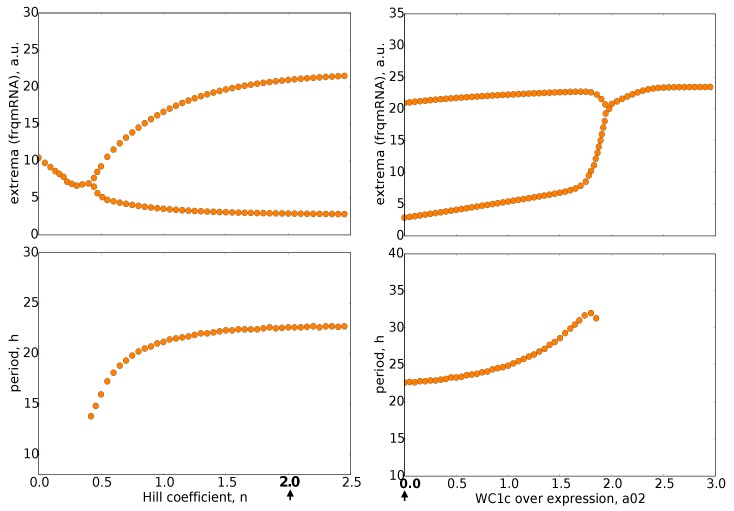
Bifurcation diagrams: The upper graphs show the maxima and minima of oscillations for varying parameters n (Hill coefficient) and a02 (WC1c overexpression). The lower graphs depict the corresponding periods. It turns out that oscillations persist for Hill coefficient n between 0.5 and 2.5, whereas overexpression of WC1 (a02) can terminate rhythms. Default parameter values are marked by bold numbers and arrows.

**Figure A2:**
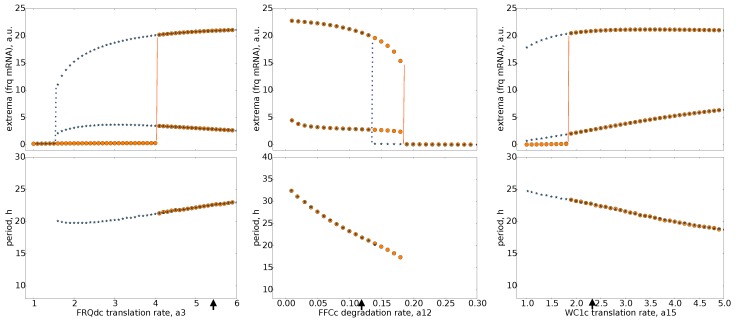
Co-existence of attractors: Bifurcation analysis of three parameters shows the co-existence of the limit cycle and steady state while the parameter is slowly varied. The values for increasing parameters are marked by orange circles. Decreasing parameters correspond to blue stars. Red lines mark jumps between steady states (low frq values) to limit cycle oscillations. The associated periods are given in the lower graphs. Default parameter values are marked by arrows.

The self-sustained oscillations were obtained in wide ranges of parameters (see Figure 4 and Figure A2). Amplitudes and periods varied smoothly with most parameters. The onset of oscillations occurred in most cases via a Hopf bifurcation, a transition from damped to self-sustained rhythms. Close to Hopf bifurcations, we found large amplitude variations, but weaker dependencies of the period on the parameters.

**Figure A3:**
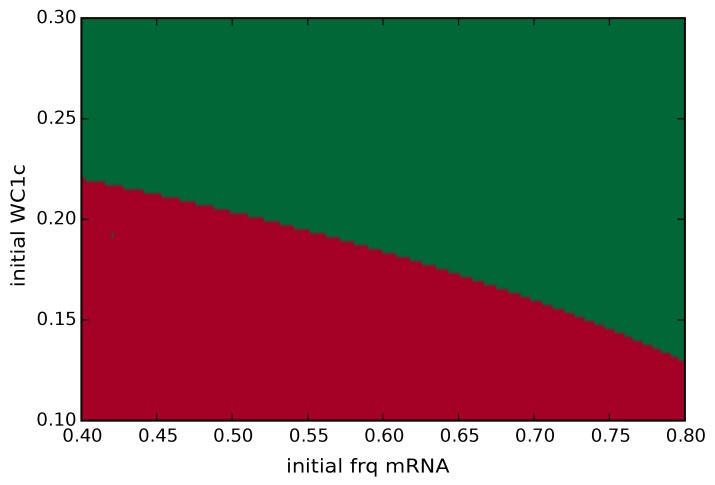
Basins of attractors: Coexisting attractors shown in Figure A2 imply distinct basins of attraction (here, a3 = 3). Small initial values of frq and wc1 (red area) approach the steady state, whereas initial conditions in green approach the limit cycle. The initial conditions of the other variables were zero.

Interestingly, for three parameters, we observed hard onsets of oscillations (compare Appendix Figure A2), i.e., the amplitude jumped abruptly. Such bifurcations were characterized by hysteresis and co-existing attractors [39,40,41] (see the basins of attractors in Appendix Figure A3). The details of these phenomena are presented in Appendix Figure A2 and Figure A3.

**Figure A4:**
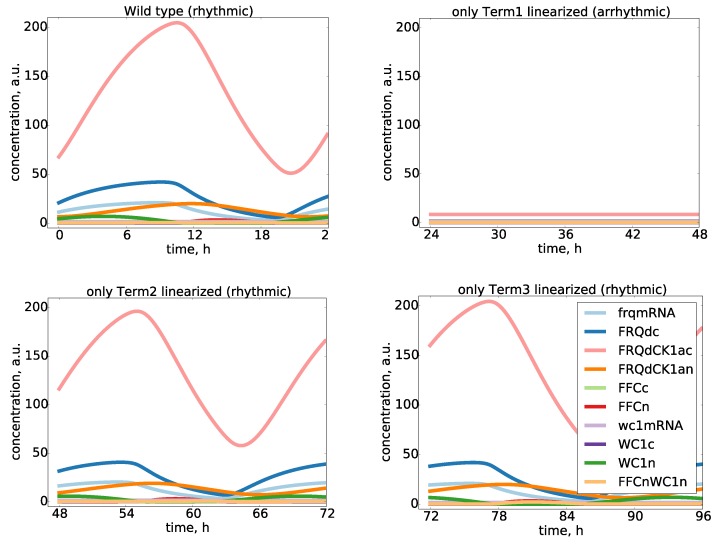
Essential and non-essential nonlinearities in the model: We linearized all three nonlinear terms and found that the Hill function of frq production (Term1) was essential, whereas the positive feedback nonlinearity (Term2) and the bilinear dimerization (Term3) were not essential since linearizations kept the rhythmicity.

Nonlinearities are necessary to obtain limit cycle oscillations. As discussed above, our model exhibited just three nonlinear terms. In order to check their relevance, we replaced the terms by their mean (termed “clamping” in [42,43]) or by a linearized kinetics. Interestingly, for two of the nonlinearities, the oscillations persisted. This implies that the dimerization and the positive feedback were not essential to obtain self-sustained oscillations. Thus, the only essential nonlinearity in our model was the Hill function describing frq transcription. Details of the detection of essential nonlinearities are provided in the Supplementary Information (Appendix Figure A4).

In summary, the developed model exhibited robust self-sustained oscillations with just a single nonlinearity and a fairly small Hill coefficient *n* = 2.

In earlier models, artificially high Hill coefficients have been used [11,12]. As in [15], we used a biologically-plausible small Hill coefficient [44]. Since there are two distinct binding sites for WCC in the frq promoter [22], a Hill coefficient of *n* = 2 was justified [45].

We can exploit now our model to discuss mutants, to analyze the underlying transcriptional switch, and to explore possible glucose compensation mechanisms.

### 2.4. Our Model Reproduced Clock Mutants

*N. crassa* has become a model organism in chronobiology since a band mutation in the ras-1 pathway leads to an overt periodic conidiation phenotype. A growth front of rhythmic conidiation and mycelium in so-called race tubes (solid culture) and gene expressions from bioluminescence signals using a frq-promoter luciferase reporter assay helped visualizing a period of about 22.5 h [46,47]. These techniques also allowed the identification of point mutations in the frq gene with altered period lengths. For example, the frq7 mutation stabilizes FRQ, leading to a longer period of about 29 h [46].

**Figure A5:**
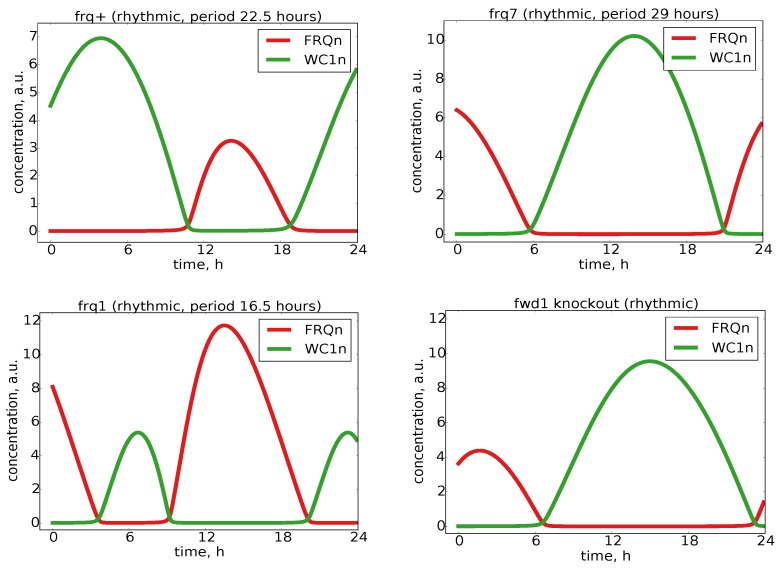
Mutant simulations: Several experimentally-observed mutants were also reproduced by our model. The corresponding modified parameters are given in Table 1.

Table 1 lists several mutant phenotypes and associated experimental findings regarding protein stability. If we adapted our model rate constants accordingly, we could simulate the phenotypes of wild-type (frq+) and shorter and longer period mutants (frq1 and frq7), as shown in Appendix Figure A5. Moreover, our model also reproduced the behavior of an FWD-1 knockout experiment demonstrating that complete turnover of FRQ was not required for the clock to run [48] (see Appendix Figure A5). This implies that the model was faithful to the experimental results, which is a good indication for any mathematical model.

### 2.5. An Inactivation-Switch Allows Self-Sustained Oscillation

It is widely known that molecular switches support limit cycle oscillations [50,51,52,53]. Moreover, the switch-like waveforms in the time series of our model discussed above indicate a switch. However, the biological mechanism of such a switch is not immediately evident.

Multiple mechanisms have been discussed that allow switch-like behavior. For example, cooperativity [54,55], zero-order ultrasensitivity [56], and multiple phosphorylations [57,58] can lead to quite steep input-output relationships [59]. Furthermore, sequestration [60] and positive feedback [61] can generate bistable switches [62,63,64,65].

**Figure 5:**
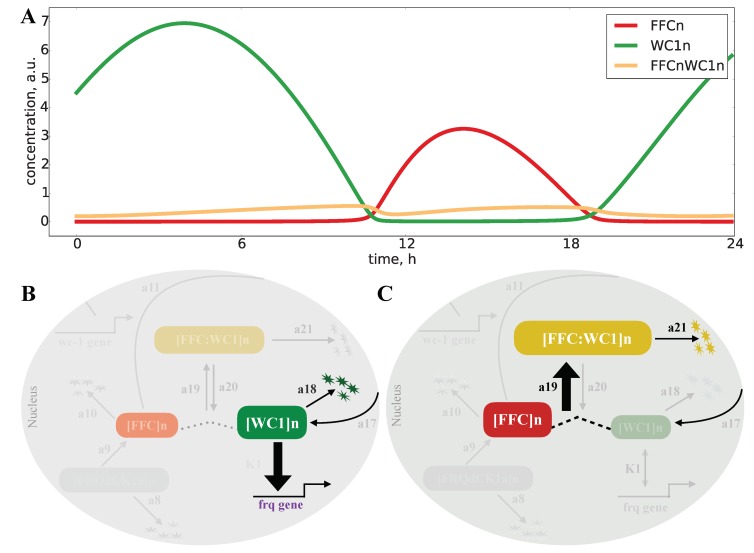
Inactivation switch in the *Neurospora* clock model: The FFCn levels (red line) are most of the time lower than the WC1n levels (green line), and only a small fraction of WC1n is bound to FFCn (yellow line) (see **A**). Below, we distinguish on and off phases. (**B**) points to an active WC1 (the thick line indicates strong binding to frq promoter) with a small degradation rate a18 (thin line). (**C**) illustrates the off state with FFC-assisted inactivation of WC1. Here, we have a strong binding of FFC to WC1 (a19 > a20) and fast degradation (a21 > a18).

Our model shows that expression of FFCn was mostly smaller than WC1n. Only a small fraction of WC1n was bound in the complex FFCWC1n (yellow line in Figure 5A). Therefore, a pure sequestration of WC1n by FFCn is unlikely. However, an inspection of the rate constants (Appendix Figure A1) revealed the underlying switch mechanism: The degradation rate a18 of the active WC1n was quite low. If the levels of FFCn grew around Time Point 10 in Figure 5A, fast binding of FFCn to WC1n (a19 > a20) allowed the sudden deactivation of WC1n (a21 > a18). A switch between active transcription to FFC-assisted fast inactivation served as the basic mechanism. Note, that these steps required neither high Hill coefficients, nor positive feedback, as in many other oscillator models [12,66].

### 2.6. Sensitivity Analysis Pointed to Inherent Glucose Compensation Mechanisms

Circadian clocks, in general, have to be robust against environmental fluctuations. Therefore, they utilize compensation mechanisms against the changes in temperature and nutrients (glucose), where changes in these environmental signals do not affect the overall clock period. There are suggested temperature compensation mechanisms [67,68,69] and glucose compensation via CSP1 [16].

**Figure 6:**
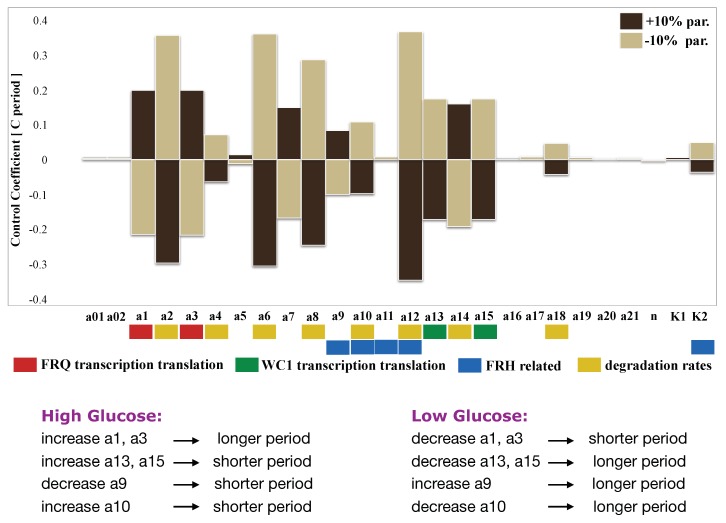
Sensitivity analysis: All parameters were changed by ±10 percent, and the resulting periods were calculated. Positive and negative period control coefficients indicate period lengthening and shortening, respectively.

Here, we study the possibilities for glucose compensation without an extra feedback CSP1 [16] using our model. We assumed that high glucose facilitates transcription and translation of FRQ and WC1. Simulations showed that faster transcription and translation rates (a1,a3) of frequency increased the oscillatory period (compare Figure 6). This was consistent with the data from the mammalian circadian clock [70,71]. With a systematic sensitivity analysis (Figure 6), we searched for putative compensation mechanisms. A promising candidate was the helicase FRH, since it has a dual role in the network: degradation of nascent mRNAs and formation of the FFC complex [27,72].

To quantify period changes for varying the parameters, we calculated the control coefficients by quantifying the sensitivity of the system. In Figure 6, we show a comprehensive control analysis of our model along the lines of [37,45,73]. All parameters were changed by ±10 percent, and the corresponding period changes were extracted from the simulations. A positive period control coefficient of 0.5 implies that a 10 percent parameter increase induced a period lengthening by five percent, i.e., about 1 h. Our analysis confirmed the well-known feature that faster degradation of frequency leads to shorter periods [74], as discussed above for the $frq^{1}$ mutant (see the yellow bars associated with increasing parameters a2, a4, a6, a8, and a10).

**Figure A6:**
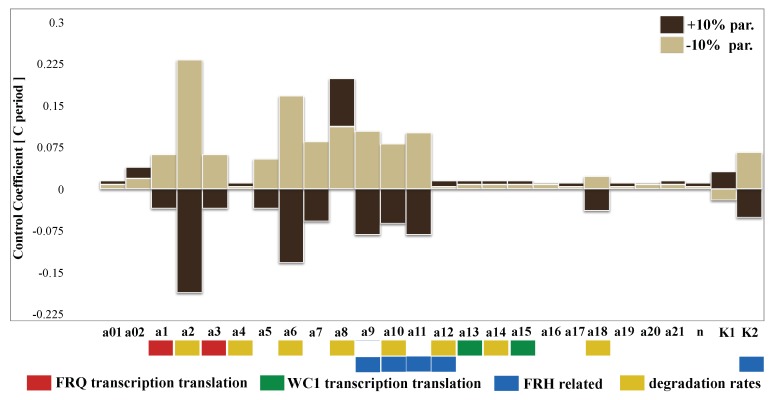
Sensitivity analysis after clamping the positive feedback: All parameters were changed by ±10 percent, and the resulting periods were calculated. Positive and negative period control coefficients indicate period lengthening and shortening, respectively (compare Figure 6).

Now, we discuss possible mechanisms to get compensation of high glucose levels. The period increase via faster frequency production (a1, a3) could be compensated by faster WC1 production (a13, a15) since the corresponding control coefficients have opposite signs. In order to study the dual role of FRH, we marked in Figure 6 the FRH-related parameters blue (a9, a10, a11, a12, and K2). It turned out that the dual role of FRH can indeed serve as a compensation mechanism as follows: Enhanced transcription due to high glucose sequesters the helicase FRH. This leads to a slower assembly of FFC (a smaller a9 value) and less stabilization of FFC (larger a10 value). According to Figure 6, these effects can compensate the period increase due to an increased frequency production. Interestingly, the glucose compensation through the dual role of FRH was lost if we canceled the positive feedback of FFCc on WC1c translation via clamping (Appendix Figure A6).

## 3. Discussion

Understanding circadian clocks quantitatively helps to explore their functional significance in different organisms [75]. In mammals, a functional clock can help to prevent diseases such as cancer, obesity, and depression [4]. To reveal the basic mechanisms of the underlying gene-regulatory network (see Figure 1), *Neurospora crassa* has been established as a useful model organism [3].

Mathematical modeling complements experimental studies and can help to uncover the underlying design principles [12,32]. A Transcriptional-Translational Feedback Loop (TTFL) serves as the generator of self-sustained oscillations with a period of about a day, but quantitative details of these delayed negative feedback [76] and supporting molecular switches [77] are debated.

The required delay of at least 6 h [78] is associated with gene expression, phosphorylation, nuclear translocation, complex formations, and epigenetic regulations [79,80]. In addition to appropriate delays, limit cycle oscillations require nonlinearities such as molecular switches [37], as studied extensively in cell proliferation and differentiation [50,62].

**Figure A7:**
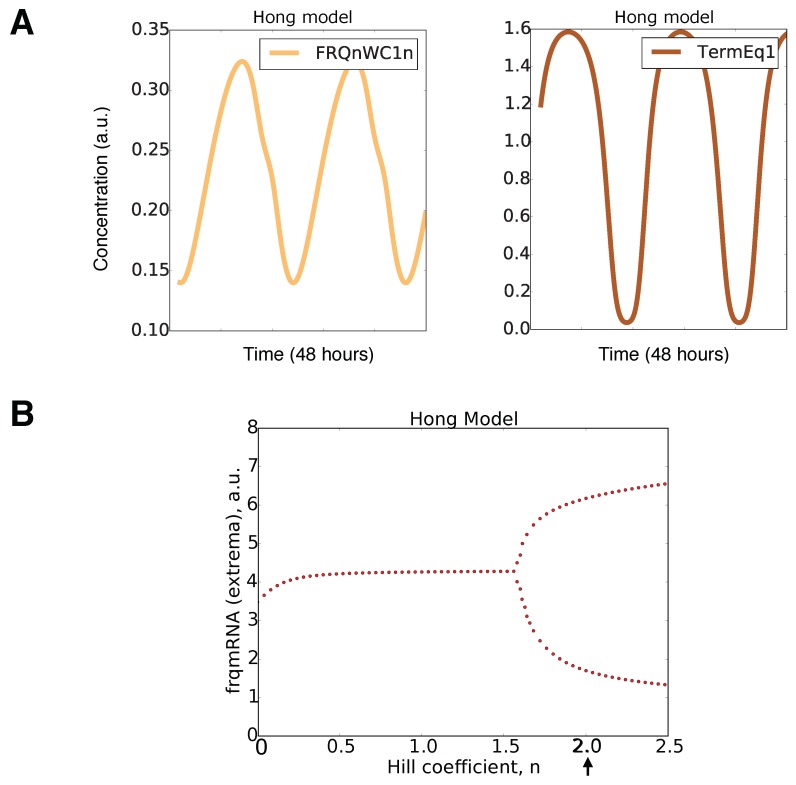
Comparisons with the Hong model [15]: (**A**) There are no double peaks of the FRQnWC1n complex and a less pronounced switch (compare Figure 3). (**B**) The Hopf bifurcation is shifted to higher Hill coefficients (compare Figure 4).

To explore the temporal dynamics of the *Neurospora* clock, we developed a mathematical model (Figure 2) that reproduces basic features of wild-type rhythms and selected mutants (Table 1). Simulations revealed spike-like waveforms and a temporal transcriptional switch (Figure 3). Appendix Figure A7A shows that harmonic waveforms and a sharp temporal switch were less pronounced in the original model [15]. There was an earlier oscillation onset in our model (compare Figure 4 and Figure A7B). Systematic clamping and linearization revealed that there was just one essential nonlinearity with a Hill coefficient of n = 2. This raises the question of how the ON/OFF switch of the frequency gene was generated without explicit ultrasensitivity or bistability.

As illustrated in Figure 5, we found sharp transitions between active transcription with slow turnover of WC1 and fast FFC-assisted inactivation of WC1. Related protein inactivation switches have been discussed in recent studies [81,82,83,84].

Our model can also be exploited to study the compensations of environmental fluctuations. It has been found experimentally that the *Neurospora* clock keeps its period almost constant at varying temperatures and glucose concentrations [67,68]. In a previous study, an auxiliary transcriptional feedback loop via CSP1 was postulated for glucose compensation [16].

Sensitivity analysis of our model (Figure 6) suggested in-built mechanisms without additional feedbacks. High levels of glucose enhance gene expression. Our model indicated that higher levels of core clock genes increased the period, as found experimentally in *Neurospora* [17,18] and in mammals [70]. Interestingly, enhanced WC1 levels decreased the period (compare Figure 6). This counter-regulation constitutes a first putative compensation mechanism.

Another glucose compensation mechanism is based on the dual role of FRH. This protein serves as an RNA helicase and as a stabilizer of the FFC complex [27,72]. Since at high glucose conditions, many more mRNAs are transcribed, FRH is partially sequestered. This implies that the FFC complex is destabilized, leading to a shortening of the period. This effect can compensate the increased period induced by enhanced transcription. Interestingly, this compensation was diminished if we turned off the positive feedback via WC1 translation [25]. This finding points to a possible role of this additional feedback regulation. Note that this feedback was quite weak in the original model [15]. We decreased the activation threshold K2 for WC1 translation to strengthen the experimentally-verified positive feedback [18,25].

In summary, we modified a model by extending the negative loop via FRH complexes and by strengthening the positive feedback. Both modifications contributed to the robustness of the model (Figure 4), the inactivation switch (Figure 5), and intrinsic glucose compensation (Figure 6). Our proposed mathematical model with 10 dynamic variables and 26 kinetic parameters reproduced many experimentally-known features. A comprehensive analysis of the model revealed some unexpected results. It turns out that there was just a single nonlinearity essential for self-sustained oscillations. The switch-like behavior of the model arose from a novel inactivation switch based of WC1 inactivation assisted by the FFC complex. This finding suggests that forthcoming experiments should be oriented towards the detailed mechanisms of WC1–FFC interactions.

Another result concerns the compensation of environmental perturbations. Sensitivity analysis of our model suggested putative in-built mechanisms to achieve glucose compensation. First, expression of the WC1 gene could counteract the effects of frequency expression on the period. Another mechanism points to possible roles of FRH and the positive feedback. Sequestration of FRH via increasing mRNA transcription leads to a destabilization of the FFC complex, which can compensate period changes in the presence of the positive feedback.

Even though our model was not quantitative in any detail, the comparison of simulations with experimental data can stimulate future investigations. The combination of a delayed negative feedback with an inactivation switch is a robust design principle that could be relevant also in other oscillatory systems such as cell cycle dynamics [85], redox oscillations [86], or somite formation [87,88].

## 4. Materials and Methods

All the simulations were performed using codes on a Spyder Python 3.4 platform. Ordinary differential equations were written into codes using the SciPy odeint library. Matplotlib library was used to generate figures. XPP-Auto was used to reproduce the bifurcation diagrams generated by Python. Codes are available upon request.

## Figures and Tables

**Table 1:** Modeling *Neurospora* clock mutants: A list of selected mutants and their properties (simulations in Appendix Figure A5).

Genotype (Name)	Genotype (Mutation)	Period (Race Tube)	Phenotype (Race Tube)	Core Clock Genes (Luciferase Reporter)	Temperature Compensation	Glucose Compensation	Proposed Effects Experimentally	Model Representation of Effects	References
$frq^{+}$ wild-type	ras1 band	22.5 h	rhythmic	oscillate	normal	normal	[FRQ]n < [WC1]n	default parameters (a7 = 0.05, a8 = 0.34, a10 = 0.10)	[46]
$frq^{1}$	Gly-Ser G482S	16.5 h	rhythmic	oscillate	normal	normal	[FRQ]n of $frq^{1}>offrq^{+}$, decreased FRQ stability, and [FRQ]n > [WC1]n	faster nuclear import rate a7 = 2.5 and increased FRQ degradation rate a8 = 0.68	[46]
$frq^{7}$	Gly-Asp G433D	29 h	rhythmic	oscillate	partially lost		increased FRQ amplitude, increased FRQ stability, and approximately [FRQ]n = [WC1]n	decreased FRQ degradation rate a8 = 0.17	[46,49]
fwd1	$fwd1^{RIP}$ COP9-signalosome	undefined	arrhythmic	oscillate			incomplete turnover of FRQ	no FFCn degradation rate a10 = 0	[48]

## Abbreviations

The following abbreviations are used in this manuscript:

*N. crassa*	*Neurospora crassa*
frq	*Frequency* gene
FRQ	Frequency protein
WCC	White Collar Complex Protein
WC1	White Collar-1 Protein
WC2	White Collar-2 Protein
wc1	*White Collar-1* gene
CK1a	Casein Kinase 1a
FRH	Frequency-interacting Helicase
FFC	Frequency FRH CK1a complex
FFCWC1	FFC and WC1 complex
FWD1	F-box/WD-40 repeat-containing protein-1
CSP1	Conidial Separation-1
a.u.	arbitrary units
ODEs	Ordinary Differential Equations
N.A.	Not Applicable
